# Strong Signature of Natural Selection within an *FHIT* Intron Implicated in Prostate Cancer Risk

**DOI:** 10.1371/journal.pone.0003533

**Published:** 2008-10-27

**Authors:** Yan Ding, Garrett Larson, Guillermo Rivas, Cathryn Lundberg, Louis Geller, Ching Ouyang, Jeffrey Weitzel, John Archambeau, Jerry Slater, Mary B. Daly, Al B. Benson, John M. Kirkwood, Peter J. O'Dwyer, Rebecca Sutphen, James A. Stewart, David Johnson, Magnus Nordborg, Theodore G. Krontiris

**Affiliations:** 1 Division of Molecular Medicine, Beckman Research Institute of the City of Hope, Duarte, California, United States of America; 2 Department of Cancer Genetics, City of Hope, Duarte, California, United States of America; 3 Department of Radiation Medicine, Loma Linda School of Medicine, Loma Linda, California, United States of America; 4 Department of Population Science, Fox Chase Cancer Center, Philadelphia, Pennsylvania, United States of America; 5 Division of Hematology/Oncology, Department of Medicine, Robert J. Lurie Comprehensive Cancer Center, Northwestern University School of Medicine, Chicago, Illinois, United States of America; 6 Division of Hematology/Oncology, Department of Medicine, University of Pittsburgh Cancer Center, Pittsburgh, Pennsylvania, United States of America; 7 Division of Hematology/Oncology, Department of Medicine, University of Pennsylvania Cancer Center, Philadelphia, Pennsylvania, United States of America; 8 Interdisciplinary Oncology Program, H. Lee Moffitt Cancer Center and Research Institute, University of South Florida, Tampa, Florida, United States of America; 9 University of Wisconsin Comprehensive Cancer Center, University of Wisconsin School of Medicine, Madison, Wisconsin, United States of America; 10 Division of Hematology/Oncology, Department of Medicine, Vanderbilt-Ingram Cancer Center, Vanderbilt University School of Medicine, Nashville, Tennessee, United States of America; 11 Department of Molecular and Computational Biology, Biological Sciences, University of Southern California, Los Angeles, California, United States of America; Indiana University, United States of America

## Abstract

Previously, a candidate gene linkage approach on brother pairs affected with prostate cancer identified a locus of prostate cancer susceptibility at D3S1234 within the fragile histidine triad gene (*FHIT*), a tumor suppressor that induces apoptosis. Subsequent association tests on 16 SNPs spanning approximately 381 kb surrounding D3S1234 in Americans of European descent revealed significant evidence of association for a single SNP within intron 5 of *FHIT*. In the current study, re-sequencing and genotyping within a 28.5 kb region surrounding this SNP further delineated the association with prostate cancer risk to a 15 kb region. Multiple SNPs in sequences under evolutionary constraint within intron 5 of *FHIT* defined several related haplotypes with an increased risk of prostate cancer in European-Americans. Strong associations were detected for a risk haplotype defined by SNPs 138543, 142413, and 152494 in all cases (Pearson's χ^2^ = 12.34, df 1, *P* = 0.00045) and for the homozygous risk haplotype defined by SNPs 144716, 142413, and 148444 in cases that shared 2 alleles identical by descent with their affected brothers (Pearson's χ^2^ = 11.50, df 1, *P* = 0.00070). In addition to highly conserved sequences encompassing SNPs 148444 and 152413, population studies revealed strong signatures of natural selection for a 1 kb window covering the SNP 144716 in two human populations, the European American (π = 0.0072, Tajima's D = 3.31, 14 SNPs) and the Japanese (π = 0.0049, Fay & Wu's H = 8.05, 14 SNPs), as well as in chimpanzees (Fay & Wu's H = 8.62, 12 SNPs). These results strongly support the involvement of the *FHIT* intronic region in an increased risk of prostate cancer.

## Introduction

The genetic complexity of prostate cancer has been well-demonstrated by independent, large-scale, genome-wide association studies that identified multiple risk loci throughout the human genome [1], [2], [3], [4], [5], [6]. These loci each only moderately increases a person's risk of the disease by up to 60% and may collectively account for over 50% of the genetic risk of prostate cancer observed in the human population. Additional risk loci remain to be discovered through meta-analysis of existing data and further study.

We recently used linkage analysis of candidate genes and subsequent association tests to implicate a 30 kb region within intron 5 of *FHIT* in prostate cancer risk [7]. The *FHIT* gene, which encodes a 16.8 kD triphosphatase, comprises 10 short exons spanning approximately 1.5 Mb. It resides at the most frequently observed fragile site in the human genome, *FRA3B* (3p14.2); and it is one of the earliest and most frequently deleted regions in multiple cancer types [8], [9]. Although deletion of the *FHIT* gene in prostate cancer tissue has not been widely reported, loss of heterozygosity (LOH) has been reported in 2 of 15 tumors through the use of microsatellite markers located in introns of *FHIT* [10]. Loss of *FHIT* was also detected in an *in vitro* model of a prostate cancer tumor cell line that was established by using HPV-18 to immortalize a normal adult human prostate epithelium cell line, followed by malignant transformation through exposure to a chemical carcinogen [11], [12]. Immunohistochemical analysis in primary cancer tissue confirmed the absence or greatly reduced expression of FHIT protein levels in tumor cells, in contrast to high levels of expression in the adjacent normal prostate epithelium [12], [13].

Although FHIT protein expression is lost or reduced in many types of human cancers [9], the mechanistic basis for the involvement of intron 5 in genetic risk of prostate cancer is not apparent. A germ-line alteration in *FHIT* that is associated with cancer risk has not been reported, possibly because of limitations of previous studies that focused only on exons, untranslated regions of mRNA, and promoters. Characteristic landmarks of a fragile region, such as aphidicolin-induced hybrid breaks, HPV16 integration sites, pSV2neo integration sites, and deletion end points in cancer cell lines, have been identified within introns of *FHIT* [14]; however, these landmarks do not overlap with the region within intron 5 that we implicated in prostate cancer risk. FHIT plays an important role in inducing apoptosis of cells responding to DNA damages caused by exposure to a variety of environmental agents, such as radiation, viruses, and toxic chemicals present in tobacco smoke and tin mines [15], [16], [17]; yet the genetic elements that control such processes have not been identified.

The evolutionary forces of mutation, natural selection, genetic drift, and recombination have shaped the pattern of variation in the human genome. Natural selection, which acts on functionally important genetic variations that result in alteration of fitness, such as adaptation to local environment and disease susceptibility, may leave specific signatures on affected loci [18], and analysis of genetic variation in populations is becoming central to understanding the function of genes [19], [20]. Screening for signatures of natural selection may help uncover novel functional elements. Therefore, we used this approach to determine whether evidence of selection could be detected within the 30 kb *FHIT* intronic region. We conducted a re-sequencing survey and analyzed linkage disequilibrium (LD) and haplotype structure in sequences from intron 5 of *FHIT* in European American, Yoruban, and Japanese populations, and several non-human primates. Based on these data, we refined the region associated with prostate cancer risk to a 15 Kb LD block and revealed strong signatures of selection in multiple human populations and, possibly, other primate species.

## Results

### Re-sequencing

Larson et al. [7] tested 16 SNPs spanning a 381 kb region within intron 5 of *FHIT* for association with prostate cancer risk and detected a significant association with one of the SNPs, rs760317. A less significant association with prostate cancer risk was found at a closely linked SNP, rs722070, located 13 kb from rs760317 on the centromeric side; no association was detected with SNPs on the telomeric side. To map the association with prostate cancer risk at high resolution and look for evidence of selection, we conducted a re-sequencing survey using 13 randomly chosen cases and controls of European-American descent. The total surveyed sequence length was 28.5 kb, excluding two un-amplifiable sequence gaps of 487 bp and 263 bp. One of the gaps contained an AT repeat and a long poly A tract, and the other contained AT and AG repeats. Two fragments of this region with lengths of 19 kb (from 134 kb to 153 kb; GeneBank Accession #AF152364) and 7 kb (from 142 kb to 149 kb internal to the 19 kb; GeneBank Accession #AF152364) were also sequenced in 16 Yoruban and 16 Japanese individuals, respectively.

Across the entire region, we identified 216 SNPs and 9 indels, ranging from 1 to 24 bp, in the 13 European-American individuals (Table S1). Within the 19 kb region, sequenced in both European-Americans and Yorubans, we found 146 SNPs and 7 indels common to both populations, 99 SNPs and 1 indel unique to Yorubans, and 19 SNPs and 1 indel unique to European Americans. Within the 7 kb region sequenced in all three populations, we detected 64 SNPs and 4 indels common to the three populations; 28 SNPs and 1 indel unique to Yorubans; 2 SNPs unique to European-Americans; 1 SNP unique to the Japanese; and 9 SNPs and 1 indel common to two populations. Indels and SNPs within long tracks of simple repeats were not included in these statistics and subsequent analyses because of the low accuracy of sequencing in these areas.

### Linkage disequilibrium (LD)

**Figure 1:**
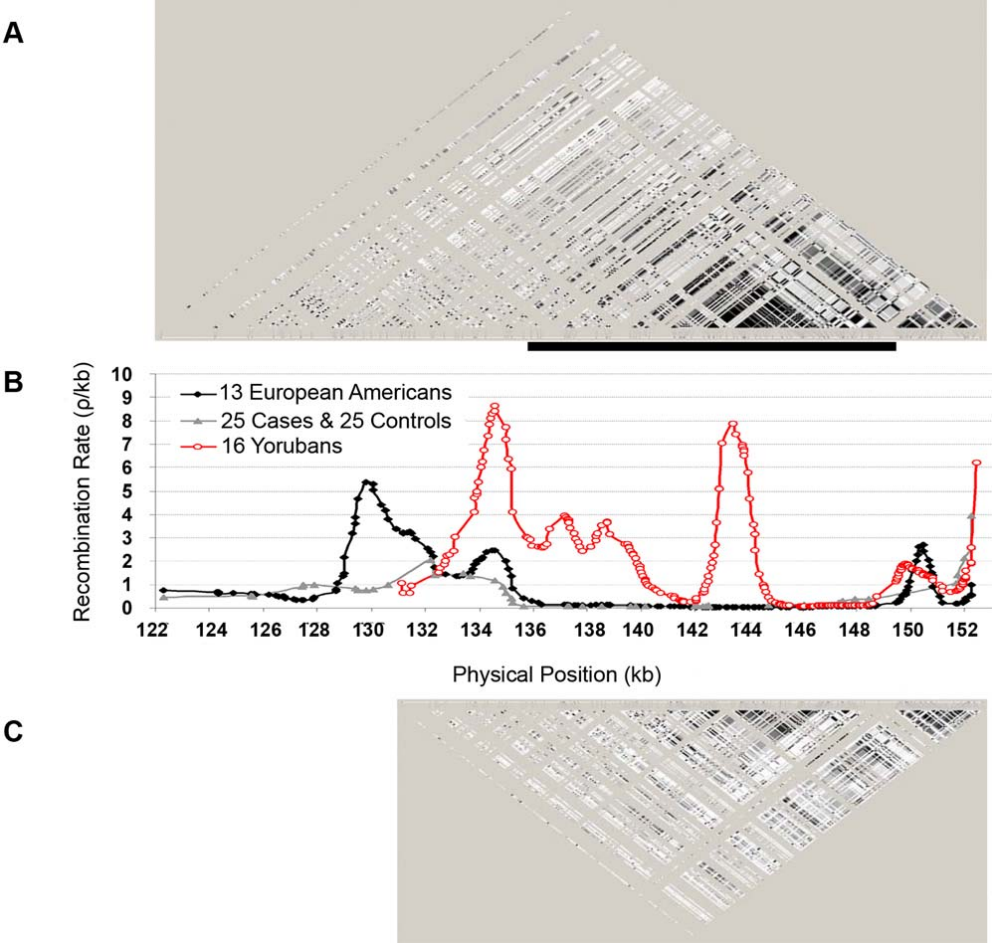
Local linkage disequilibrium (LD) structure and recombination rates (based on SNPs with minor allele frequencies > = 0.05 in a 30 kb region of *FHIT* intron 5). A. Graphical representation of pair-wise r^2^ (from 0 to 1 represented with gray scale from white to black) calculated and visualized using Haploview for 13 European Americans. B. Recombination rates (Rho) calculated using rhomap for Yorubans (red line, with SNP positions represented by open circles) and European Americans (black line, with SNP positions denoted by solid diamonds) based on sequencing data. The grey line with solid triangles was based on genotyping data on 51 SNPs from 25 cases and 25 controls (European Americans). C. Graphical representation of r^2^ using for 16 Yorubans. A solid black bar represented a 15 kb LD block in the European American.

We calculated pair-wise r^2^ based on SNPs with a minor allele frequency greater than 0.05 (196 SNPs for the 13 European-Americans samples and 178 SNPs for the 16 Yorubans samples) using Haploview (Fig. 1A and C) and recombination rates with hotspots using rhomap (Fig. 1B). Consistent with previous reports [21], [22], we observed much less LD in the African sample, although the pattern of LD was otherwise similar between the populations. The most noticeable difference between the two populations was a 15 kb LD block in the European American population which was disrupted by much higher background recombination rate and at least one recombination hotspot in the Yoruban population (Fig. 1B). We selected 48 SNPs from the re-sequencing survey and three SNPs published in Larson et al. [7] that represented the basic LD structure and genotyped these SNPs in all case and control samples to evaluate their association with prostate cancer.

### Association Tests

**Figure 2:**
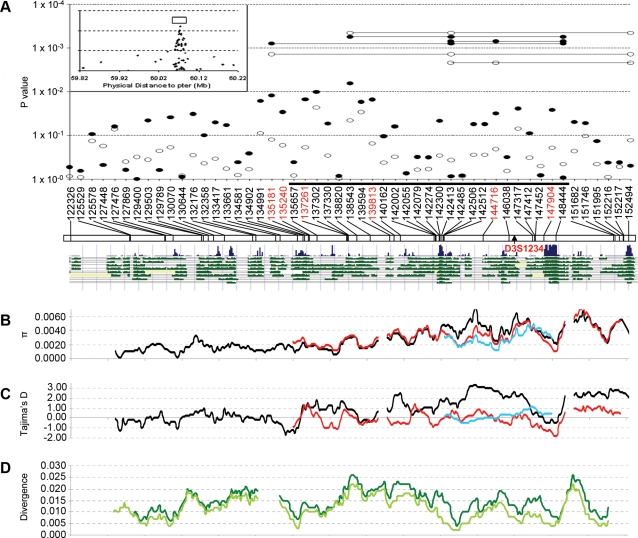
Co-localization of SNP association and natural selection. A. Association tests of single SNPs and haplotypes. Individual SNPs were anchored on an UCSC Genome map with Multiz alignment and conservation of vertebrates (v166; http://genome.ucsc.edu) for the 30 kb region. The region was represented with an open bar in an inset at the upper left corner depicting single SNP tests surrounding a broader 381 kb region. An arrow pointed to the microsatellite marker, D3S1234, exhibiting the strongest linkage signal in the original study. A solid black bar corresponds to the 15 kb LD block in the European American. Tests on allele frequency for individual SNPs are denoted by circles (open for all cases and black for 2 IBD cases). Tests for risk haplotyes are represented by circles linked with lines. SNPs highlighted in red are in strong LD (r^2^>0.9) with each other. B. Nucleotide diversity (π) calculated for Yorubans (red line), European Americans (black line), and Japanese (blue line). C. Tajima's D calculated for Yorubans (red line), European Americans (black line), and Japanese (blue) using SLIDER. D. Diversity between human and chimpanzee sequences (dark green line including SNPs in humans and light green line excluding SNPs in humans).

We performed association tests on individual SNPs and haplotypes of SNP combinations. Since the original linkage data predicted a recessive model [7], we hypothesized that the subgroup of cases that shared 2 alleles identical by descent (IBD) at this locus with their brothers (2 IBD cases) would be the major contributors to the observed genetic signal. Therefore, we compared SNP frequencies in controls against all cases and 2 IBD cases (Table S2). Significant association (cutoff *P* = 0.05, not corrected for multiple testing) was observed for several SNPs and maximized at 137302 (rs9814915, Pearson's χ^2^ = 5.16, degrees of freedom (df) 1, *P* = 0.0231) for all cases (single open circles in Fig. 2A) and 138543 (rs760317, Pearson's χ^2^ = 7.42, df 1, *P* = 0.0064) for the 2 IBD subgroup (single black circles in Fig. 2A).

Screening for haplotype association for all three-SNP combinations revealed that the strongest association of prostate cancer risk was with a haplotype defined by SNPs 135181, 142413, and 152494 in 2 IBD cases (haplotype G-G-T was enriched in 2 IBD cases, χ^2^ = 9.73, df 1, *P* = 0.0018, Table S3) and SNPs 138543, 142413, and 152494 in all cases (haplotype A-G-T was enriched in all cases, χ^2^ = 13.72, df 1, *P* = 0.00021, Table S3). Adding any other single SNP to the combination did not increase the association with prostate cancer risk, while omitting any SNP in the combinations significantly reduced the signal (data not shown). Consistent with a recessive model, samples that were homozygous for the risk haplotype were significantly enriched in cases as compared to controls.

Interestingly, both SNP combinations identified in all cases and 2 IBD cases included SNPs 142413 and 152494, which individually exhibited very limited association with prostate cancer risk. SNP 152494 was located within a highly conserved non-coding sequence, and SNP 142413 was located within 100 bp of another highly conserved non-coding sequence (Fig. 2A). Neither SNP was in strong LD with any other SNP genotyped in cases and controls. However, SNP 135181 was in strong LD with SNP 138543 (r^2^ = 0.86) and several other genotyped SNPs, 135240, 137261, 139813, 144716, 147904 (r^2^ ranging from 0.87 to 0.97, highlighted SNPs in Fig. 2A). Therefore, these other SNPs also exhibited a compelling association to prostate cancer risk in combination with SNPs 142413 and 152494 (SNP combinations represented by open circles linked with a line in Fig. 2A and Table S3). An additional 21 SNPs were known to be strongly linked to 135181 based on sequencing data. Among all the SNPs linked to 135181, only 147907 was located within a highly conserved sequence (Fig. 2A). Therefore, SNP 147907 may be a likely candidate for causality.

A SNP, 148444, located within a highly conserved sequence, showed the highest LD (r^2^ = 0.37) with SNP 152494 among all genotyped SNPs. Replacing SNP 152494 with 148444 in the three-SNP combinations also defined a haplotype for which homozygotes were especially enriched in cases (Table S3). Consistent with a recessive model, we found the strongest association with homozygotes of the risk haplotype in 2 IBD cases (SNP combinations represented by black circles linked by a line in Fig. 2A)—even stronger than most combinations with SNP 152494.

### SNPs Underscoring a Signature of Natural Selection in Humans Are Associated with Prostate Cancer Risk

To discriminate SNPs that might contribute functionality among the SNPs showing strong association with prostate cancer risk, we used re-sequencing data from European-American, Yoruban, and Japanese populations to search for signatures of natural selection, in addition to conservation, within the 28.5 kb region. Several key statistics were calculated using SLIDER. We compared these population parameters in the *FHIT* interval to those obtained in the HapMap ENCODE Sequencing Project, in which 10 regions of 500 kb from various chromosomes in four human populations were sequenced in their entirety. These ENCODE data provided a reasonable control for the genome-wide distribution of population-specific statistics. To confirm the statistics observed in the 13 randomly-selected cases and controls, we also sequenced a 2 kb region containing the maximum Tajima's D value in 14 CEPH individuals. Because the ENCODE data do not provide genotypes on indels, we also excluded indels from our *FHIT* region in population analyses and comparisons.

We observed a striking increase of nucleotide diversity that spanned multiple LD blocks for the three human populations (Fig. 2B). The maximum π was 0.0072 (0.0065 for the 14 CEPH individuals), 0.0077, and 0.0049 for European-American, Yoruban, and Japanese populations, respectively, compared to an average of 0.00071 (0.000071 to 0.0055), 0.00074 (0.00013 to 0.0046), and 0.00076 (0.00013 to 0.0050) within the 5 Mb ENCODE regions. Therefore, the maximum π observed within the 28.5 kb region in intron 5 of *FHIT* exceeded the maximum π observed from the 10 ENCODE regions for both European-Americans and Yorubans (p<0.0060 for both populations).

We also detected a significant increase of Tajima's D in the European-American population (Fig. 2C). The 1 kb window of maximum Tajima's D (3.31, *P* = 0.003 assuming standard neutral model I; *P* = 0.006 assuming neutral model II; *P* = 0.021 assuming neutral model III) corresponded to the window of maximum π. In the 14 CEPH individuals, the same window exhibited a Tajima's D of 3.11. Only one small ENCODE region, less than 0.6% of all ENCODE regions, displayed a higher maximum Tajima's D. A significant Fay & Wu's H value (8.05 for 14 SNPs, *P* = 0.0067, assuming a standard neutral model I) was detected for the same window in the Japanese population. A 14-SNP sliding window analysis for all ENCODE regions in the Japanese population found 237 of 6265 windows with a Fay & Wu's H value greater than 8.05 (*P* = 0.038).

To determine if the greater nucleotide diversity was due to an increase in the local mutation rate, we evaluated the nucleotide differences in the 28.5 kb sequenced region by comparing one human sequence with one chimpanzee sequence retrieved from the UCSC genome browser. For each 1 kb window across the 28.5 kb region, the divergence between the human and the chimpanzee sequences ranged from 0.004 to 0.026 and averaged 0.0145 (Fig. 2D). For the 1 kb region with the maximum π in the European-American population, we observed a divergence of 0.0150. Divergence values for the adjoining 5 and 10 kb were 0.0137 and 0.0149, respectively. These statistics were only slightly higher than the average for the chimpanzee genome (0.0123 [23]). When SNPs that are observed in human populations were excluded, the divergence was significantly reduced (ranging from 0.002 to 0.022 and averaging 0.0112), especially within the region that showed high nucleotide diversity in humans (Fig. 2D). These observations excluded a higher local mutation rate as a major cause of greater diversity in human populations.

Two SNPs, 144716 and 144552, within the 1 kb window that showed the maximum signal of natural selection, were in strong LD with 135181 and demonstrated a comparable level of association to prostate cancer risk in combination with SNPs 142413 and either 152494 or 148444 (Table S3). The region from 142 kb to 149 kb displayed significantly higher nucleotide diversity among European-Americans than Yorubans, in contrast to surrounding regions and the vast majority of the human genome, in which Yoruban diversity is generally similar to or higher than European-American diversity (Fig. 2B). This region also encompassed three SNP combinations: 142413, 144716 or 147904, and 148444, each residing within a sequence under natural selection and jointly delineating the putative risk haplotype. This overlap of selection and significant association signals implicated co-evolution and interactive functions among the sequence modules in their involvement in prostate cancer risk.

### Signatures of Selection in Non-Human Primates

Sequencing data in the same 1 kb window in common western chimpanzees and bonobos also revealed potential natural selection. Although the chimpanzee sequence possessed a completely different collection of SNPs compared to the human sequence, the haplotype distributions exhibited a pattern similar to that of the Japanese population: predominantly one haplotype with extremely high frequencies of the derived allele for multiple SNPs (Tajima's D = −1.81, Fu and Li's D = −3.02, π = 0.0015). A significantly high Fay & Wu's H (8.62 for 12 SNPs, *P* = 0.0001 assuming the standard neutral model) suggested a hitchhiking effect under a recent positive selection. For the bonobo sequence, two rare SNPs, each observed only once in the 6 individuals, and no fixed nucleotide change were present in the 1 kb window (Tajima's D = −1.45, FuLi D = −1.72, π = 0.00034) compared to chimpanzee sequence.

Three subspecies are recognized among common chimpanzees based on their geographic distribution: *Pan troglodytes verus (Ptv)* in western Africa, *Pan troglodytes troglodytes (Ptt)* in central Africa, and *Pan troglodytes schweinfurthii (Pts)* in eastern Africa. Previous studies suggested distinct demographic histories for the three subspecies, resulting in a slightly positive average value of Tajima's D for western chimpanzees (*Ptv*) and a significantly negative average value of Tajima's D for central chimpanzees (*Ptt*). To establish a genome-wide distribution of population statistics for the common chimpanzees, we retrieved and reanalyzed sequence data from two previous studies: 50 intergenic regions (Genebank acc.# AY276396 to AY277244) sequenced in 17 common chimps (6 *Ptv*, 5 *Ptt*, 2 *Pts*, and 4 unknown) [24] and 10 non-coding regions sequenced in 14 central chimpanzees [25] from the NCBI database (Table 1). The statistics observed in the 1 kb target region (Tajima's D = −1.81, Fu & Li's D = −3.02) placed it among the lowest of genome-wide distributions. Sequencing data for a larger number of primate individuals that is analyzed separately for each subspecies will be required to evaluate the effect of natural selection with higher confidence. Nevertheless, these preliminary data are consistent with signatures of selection in a primate species other than humans.

**Table 1:** Summary statistics of common chimpanzees based on previously published sequencing data.

Subspecies Name (# of Individual)	# of Regions (# of Chromosome)	Total Sequenced Length (kb)	Tajima's D			Fu & Li's D			Reference
			Average	Max.	Min.	Average	Max.	Min.	
Ptv. (6)	50 (17)	23	0.093	1.67	−1.63	0.17	1.26	−1.95	[24]
Ptt. (5)	50 (17)	23	−0.42	1.30	−1.90	−0.41	1.15	−2.22	
Combined (17)	50 (17)	23	−0.99	1.00	−2.01	−1.07	1.27	−3.61	
Ptt (14)	9 (8)	19	−0.95	0.85	−2.12	−0.36	1.44	−2.60	[25]

## Discussion

Previous linkage and association studies identified an approximately 30 kb region associated with prostate cancer risk. In this report, detailed analysis of local LD structure and additional association tests refined the maximum signal to within a 15 kb region, possibly involving a haplotype defined by three or more SNPs within sequences under strong evolutionary constraint.

Evidence of both association and selection supported important and interactive functions for sequences within the 15 kb intronic region of *FHIT*. The risk haplotypes defined by major alleles of several SNPs in combinations were not in complete LD with any single SNP discovered in the 28.5 kb region and exhibited much stronger associations with prostate cancer than any single SNP tested. Among the 9 SNPs that delineated risk haplotypes, four (142413, 147904, 148444, and 152494) were located within or near sequences that are highly conserved among mammals; and one (144716) was located within a sequence that exhibited significant and distinct signals of natural selection within diverse human and primate populations. The alleles of 5 SNPs (142413, 135181, 137261, 138543, and 144716) in risk haplotypes were ancestral. The alleles of both SNPs 148444 and 152494 in risk haplotypes were derived and reached very high frequency (>0.8) in all three human populations tested; therefore, they appeared to be under positive selection, especially in the Yoruban population. For example, SNP 148444 overlapped with 1-kb windows of minimum Tajima's D (−1.804 for 10 SNPs) and elevated Fay and Wu's H (9.31 for 17 SNPs) in the Yoruban population.

Levin et al. [26] recently reported an inverse association of prostate cancer risk to the SNP, rs760317 (138543), described in our original study [7]. The authors attributed the association of the “flipped” allele (G instead of A) to (i) a high minor allele frequency of rs760317, (ii) an unidentified additional causal SNP of relatively low linkage disequilibrium with rs760317, and (iii) no consideration of the interaction between the two in their analysis model. In the current study, we identified two additional SNPs, 142413 and 152494 or 148444, that interact with either SNP 138543 or a SNP in very high LD with 138543, such as 144716, that determined the risk of prostate cancer. Pairwise LD measurements among the three interacting SNPs were indeed very low (r^2^<0.3 for all possible pairs), consistent with the hypothesis originally proposed by Lin et al. [27] to explain a flipped association.

Detection of signatures of natural selection has been proposed to map genes and regulatory elements involved in human diseases [18], [28]. In this paper, we used evidence of natural selection to infer functionality of an intronic region implicated in prostate cancer [7]. Since we detected strong signals of both positive and balancing selection within the same region for different human and non-human primate populations, chance and demographic history alone can not fully explain our observations. To control for the effect of demographic history, we confirmed high Tajima's D and π in the same individuals which have been sequenced in the ENCODE Sequencing Project and provided a genome-wide background. Therefore, natural selection presents a plausible explanation for the non-random distribution of SNP genotypes existing in the data.

Population genetics in this region suggested that diverse selective forces may have been acting on different populations of humans and primates. It is, therefore, intriguing that the *FHIT* gene is known for its responsiveness to environmental factors, such as smoking [15] and radiation exposure [17], and mediates cell survival or apoptosis [9]. We compared synonymous and nonsynonymous changes in the *FHIT* coding region between the human and the chimpanzee and found 4 synonymous and 2 nonsynonymous changes within its 441 bp coding region. Both nonsynonymous changes altered the chemical properties of amino acids involved, implying that *FHIT* might be one of the fast-evolving genes subjected to positive selection.

Conventional association studies have largely focused on known coding sequences, which account for only approximately 1.5% of the human genome. However, recent studies have revealed large populations of previously unknown RNA transcripts, most of which are non-coding, within introns and intergenic regions [29], [30]. Many of these transcripts are involved in tumorigenesis [31] including prostate tumor differentiation [32]. Multiple independent studies have also confirmed the role of non-coding regions on 8q24 in susceptibility to prostate cancer [2], [33]. Within the region we implcated in prostate cancer risk, a genome-wide effort to predict conservation of RNA secondary structure using the computer program, EvoFold [34], detected a 61-bp conserved structure surrounding the SNP 148444. Whether such elements within intron 5 locus convey prostate cancer risk through alteration of *FHIT* expression/function or through unrelated intronic functional elements remains to be investigated.

## Materials and Methods

### Study Subjects

The case and control samples have been described previously [7]. The study and the use of the tissues have been approved by institutional review board at each participating site. Informed written consent was obtained from all participants. Briefly, DNAs from 200 unrelated patients of European descent affected with prostate cancer and 143 controls of matched ethnicity were used in the current study. Informed consent was obtained from all participants. In addition, DNA from 14 CEPH (European American), 16 Yoruban (African), and 16 Japanese individuals was obtained from the Coriell Cell Repositories. Samples from the 14 CEPH individuals, 8 of the Yorubans, and 8 of the Japanese had been re-sequenced in the HapMap ENCODE Resequencing Project.

We obtained the primate DNA panel (PRP00003) from the Coriell Cell Repositories. The sample included one individual from each of the following species: common chimpanzee, bonobo, gorilla, Sumatran orangutan, pigtailed macaque, rhesus monkey, black-handed spider monkey, common woolly monkey, red-chested mustached tamarin, and ring-tailed lemur. We also obtained DNA of another 12 unrelated common western chimpanzees (NS03622, NS03623, NS03639, NS03641, NS03650M NS03656, NS03660, NA03450, NG06939, NS03489, NS03610, and NS03659; personal communication, W. Winckler, The Broad Institute, Cambridge, MA) from the Coriell Cell Repository, as well as DNA from five additional unrelated bonobo individuals (identifiers available on request).

### SNP genotyping

Genomic DNA was extracted as described previously [7]. SNP genotypes were obtained and critical SNPs were confirmed using a combination of ABI SNaPshot™ genotyping on an ABI377 DNA sequencer, Sequenom iPLEX SNP typing on a MassARRAY system, and sequencing on ABI3130xl and ABI 3730 platforms.

### Re-sequencing

Genomic DNA was amplified using overlapping PCR primers and re-sequenced using PCR and internal primers. SNPs were detected using PolyPhred 4.0 [35] and Consed [36]. To minimize the false negative rate, we used a low -score setting of 50 to tag all possible SNPs and inspected each SNP manually to verify the accuracy of sequence assignments. Indels were recorded through manual inspection.

### Statistical Analyses

We used Haploview [37] to perform χ^2^ tests of Hardy-Weinberg equilibrium (HWE) for each marker genotyped in cases and controls and found no extreme deviation. We also used Haploview to calculate and visualize r^2^ between each pair of markers (minor allele frequency, MAF, ≥5%), and to compare allele frequencies of cases and controls. Recombination rates were calculated using rhomap [38] with 10000000 runs and 1000000 burn-ins. Screening for individual haplotype association of 3 SNP combinations was achieved using UNPHASED [39]. Significant SNP combinations were verified using an online Pearson's chi-square test [40] and Odds Ratio test [41] on haplotype (inferred by PHASE 2.1 [42], [43]) frequencies and genotype frequencies comparing risk haplotype against all other combined, and a permutation test for “Haplotypes in Blocks Only” implemented in Haploview.

We used an online program, SLIDER (http://genapps.uchicago.edu/slider/index.html), to obtain several population statistics, including the average pair-wise difference, π [44], summaries of allele frequencies, Tajima's D [45], Fu and Li's D [46], and Fay and Wu's H [47]. A window of 1000 bp and increment of 100 bp were applied for all analyses unless otherwise indicated.

### Model Simulations

We tested the significance of observing a given statistic by simulating sample sets under neutral models using MS, as described by Hudson [48]. The genome average of positive Tajima's D values reported for non-African populations suggests that models that include population structure and reduction in population size may be compatible with data displaying large positive deflections of D (see Results) [49]. Therefore, we considered three neutral models: a standard neutral model (neutral model I, average Tajima's D = −0.08); a neutral model assuming a population structure of four subpopulations of equal size with migration parameter, 4N_0_m = 0.7 (neutral model II, average Tajima's D = 0.4); and a neutral model assuming a recent bottleneck, in which a population was reduced to 0.35 of its original size at T_2_ = 0.0375 in units of 4N_0_ generations, followed by a population expansion starting at T_1_ = 0.000375 and reaching its current size of 1.5 times its original size (neutral model III, average Tajima's D = 0.4). Sample sets were always simulated with the same fixed number of segregating sites as observed in the region being simulated. A Bonferroni correction was applied whenever multiple windows were considered.

Sample sets of natural selection models were simulated using SelSim [50], with a fixed number of segregating sites as the DNA segment of interest. Population statistics were calculated using SLIDER as described above.

## Supporting Information

Table S1 SNPs identified within the sequenced region of *FHIT* intron 5
(0.05 MB XLS)

Table S2 Association tests for single SNPs
(0.03 MB XLS)

Table S3 Association tests for SNP combinations
(0.05 MB XLS)
